# Use and Abuse of Digital Devices: Influencing Factors of Child and Adolescent Neuropsychology

**DOI:** 10.3390/clinpract13060119

**Published:** 2023-10-27

**Authors:** Carola Costanza, Luigi Vetri, Marco Carotenuto, Michele Roccella

**Affiliations:** 1 Department of Sciences for Health Promotion and Mother and Child Care “G. D’Alessandro”, University of Palermo, 90128 Palermo, Italy; 2 Oasi Research Institute-IRCCS, Via Conte Ruggero 73, 94018 Troina, Italy; 3 Clinic of Child and Adolescent Neuropsychiatry, Department of Mental Health, Physical and Preventive Medicine, University of Campania “Luigi Vanvitelli”, 81100 Caserta, Italy; marco.carotenuto@unicampania.it; 4 Department of Psychology, Educational Science and Human Movement, University of Palermo, 90128 Palermo, Italy; michele.roccella@unipa.it

The impact of technology on human life is significant, touching various aspects such as communication, economy, education, medicine, industry, and even ecosystems. A key advantage of technology is its ability to break down physical barriers, enabling collaboration across geographies and areas of expertise. The educational landscape has been transformed by e-learning, which has revolutionized training methods, while healthcare has advanced using modern medical equipment and telemedicine.

Technology has also shaped cultural and societal evolution, influencing communication, interaction and knowledge sharing. Social media’s irruption has redefined the dynamics of personal relationships [1], but it is important to recognize that a digital divide still exists, separating different generations and regions worldwide.

Nevertheless, excessive technological interconnectivity can give rise to psychological problems, such as social isolation, anxiety and depression [2]. Individuals may show a propensity to depend on digital devices and computer networks, with consequent negative impacts on their psychophysical well-being and potentially compromise their social interactions [3].

To address the risks deriving from technological hyper-connectivity, it should be important to examine and evaluate the effect of new technologies on the population, especially on digital natives born and raised in the digital age. The term was coined by Marc Prensky in 2001 to describe those born between the late 1980s and early 2000s [4]. Digital natives are exposed from childhood to smartphones, tablets, and applications, using technology as a pervasive tool to communicate, learn, and entertain themselves. Early exposure to various digital platforms makes them multitask and constantly connected, preferring technology to social communication and interaction. Accustomed to the promptness of information, they tend to require immediate responses and may need more tolerance for the slowness of traditional processes [5]. However, the concept of digital natives is a matter of debate, as their technological competence can vary even within the same age group. Furthermore, the effect of technology on psychophysical development and education is still a topic of research and discussion [6].

“Digital disorders”, also known as Internet-use disorders, represent a category of disorders that involve mental health and can arise from the abuse of or dependence on digital technological devices, such as smartphones, computers and social media. It is worth highlighting that the term “digital disorders” has not yet obtained official recognition as a category of mental disorders in the Diagnostic and Statistical Manual of Mental Disorders (DSM) or in the International Classification of Diseases (ICD). However, mental health specialists are beginning to take these issues seriously, conducting studies to evaluate the impact of excessive technology use on individuals’ overall well-being [7].

Some of the main digital pathologies that should be interesting to evaluate are the following:

Internet Addiction Disorder (I.A.D.): Internet Addiction Disorder is characterized by an excessive level of interaction with the Internet, showing a relationship of dependence and dominance, accompanied by changes in mood, the inability to manage the time spent connected to the network, withdrawal symptoms, conflicts, and risk of relapses. This concept was introduced by the American psychiatrist Ivan Goldberg [8], who adopted the framework of pathological gambling as a reference model. Internet addiction is defined as a form of “compulsive use of technology”, leading to significant negative consequences in individual life. The main symptoms outlined are an increased desire to spend more time in using the network and an increase in the frequency of connections [1]. Individuals affected by this disorder tend to spend significant periods online, neglecting their daily obligations, social interactions and other essential activities. We can also observe a marked weakening of interest in any activity that is not connected to Internet use. In addition, states of agitation, depression and anxiety symptoms, and obsessive thoughts or dreams related to virtual activities develop when the individual is disconnected [2].

Furthermore, the inability to interrupt or reduce the use of the network is noted despite self-awareness of the problems and their related social, psychological or physical consequences [9]. The psychological and physical changes produced in the individual who becomes dependent on the Internet are loss or impoverishment of interpersonal relationships, changes in mood, alteration of time perception, a tendency to replace the real world with a virtual place, real physical symptoms such as carpal tunnel, widespread neck, back pain and vision problems. From a cognitive–behavioral point of view, the following aspects can be observed in people developing Internet addiction: dysfunctional thoughts about themselves and others, subjective feelings of inadequacy, insecurity, low self-esteem and relational problems, mood disorders, anxiety and impulse control [2].

Gaming addiction: also known as “video game addiction” or “gaming disorder”, represents a pathological behavior model characterized by excessive and compulsive use of video games, with consequent negative impacts on the social, psychological and physical sphere of the individual. Online gambling disorder, for example, includes an addiction to Internet gambling that can lead to financial, psychological and social problems. Consider, for example, the phenomenon of online poker or sports betting [10].

Social media addiction: it refers to compulsive and obsessive addiction to social media and online communication platforms such as Facebook, Instagram, Twitter and others [11]. Some typical signs and symptoms include compulsive social media use, sleep disorders, and social exclusion. The excessive use of social media can lead to a withdrawal from real social interactions favoring instead virtual connections. The individual could develop insecurity and inadequacy feelings and reduced school or work performances due to the time spent on online updates and interactions. They may experience significant mood changes based on the virtual audience’s perception of feedback (such as likes, for example) [12].

With “Problematic Social Media Use” (PSMU), we refer, however, to a pattern of social media use that becomes problematic or excessive, causing negative impacts on the individual’s daily life and well-being. This term is often used to describe obsessive and compulsive behavior related to the use of social media that can develop into an addiction [13].

Likemania is an informal term describing obsessive and compulsive behavior related to seeking appreciation or “likes” on social media. The individual is highly focused on obtaining a high number of “likes” or appreciations on posts, photos or content shared online, and they may be profoundly influenced by the followers’ reactions and constantly seeks confirmation and approval through “likes”, with excessive behavior in updating and sharing content to receive positive feedback.

Followermania is an informal term used to describe obsessive and compulsive behavior related to obtaining a high number of “followers” on social media. This term refers to the behavior of people who, in order to increase the number of followers or “accounts” subscribing to their channel or following their page, resort to excessive, sometimes risky behaviors, such as purchasing fake followers or using other questionable methods [12].

“FOMO”: An acronym for the English expression “Fear of Missing Out”, literally “fear of being left out” [14], it indicates a form of social anxiety characterized by the desire to continuously stay in contact with other people’s activities. There is a compulsive worry about missing opportunities for social interaction and the constant fear that other people will have rewarding experiences when you are not present or directly involved. FOMO involves the desire, which can become obsessive, to continuously monitor what friends publish on social networks to stay up to date. Psychological dependence on being “online” causes anxiety when you feel disconnected and excluded [15].

Vamping: It is a term used to describe the behavior of people spending much time interacting with social media, surfing the Internet or using electronic devices at night, often to the detriment of sleep and regular rest times [16]. The term is often associated with people who become “hooked” on these activities compulsively, making it challenging to maintain a healthy sleep–wake rhythm, with frequent awakenings at night to check notifications and go back online [17].

Nomophobia: From No Mobile Phone Phobia [18], it is a term recently introduced in the Anglo-Saxon world, which designates the uncontrolled fear of remaining disconnected from the mobile telephone network. Therefore, it can be translated as the “obsessive fear of not being reachable by cell phone”. Those suffering from it can experience anxiety, dizziness, sweating and panic attacks in the event of a lack of mobile network or a cell phone that is out of order. Nomophobia has increasingly become relevant with the increasing spread of mobile devices and communication technologies. The constant connection to the Internet and the dependence on mobile devices have led many individuals to develop an “attachment” to their phones [19].

Hikikomori: The Japanese psychiatrist Tamaki Saito coined the term in 1998 when he realized the symptomatic similarity of an ever-increasing number of adolescents characterized by lethargy, lack of communication and marked social isolation [20].

The symptoms recall the social withdrawal of pervasive developmental disorders, which impact social integration. It is considered a form of voluntary social exclusion, a youthful rebellion against traditional Japanese social culture, manifested by adolescents living secluded, often in a room, without external contacts [21]. It would be interesting to examine in what terms virtual experiences can satisfy the emotional and relational needs of the individual, inducing a process of replacement of the physical environment with the media one. It would be helpful to provide information for developing preventive strategies and interventions to prevent possible risks in young people’s mental and social health in the digital age.

Much evidence in scientific literature has highlighted how abuse or distorted use of new technologies can lead to a decrease in the physical or psychological well-being of children and adolescents. Therefore, today clinicians treating children and adolescents cannot underestimate during their evaluation a careful analysis of digital health. New studies will be necessary to better define these new nosographic entities related to digital environments and to outline new diagnostic and treatment protocols in order to prevent negative consequences due to an inappropriate and uncontrolled use of new technologies.

We believe that this Special Issue contributes to the enhancement of further studies on child problems related to Internet use and abuse.
